# Supplementary figures and images for: Quantitative evaluation of therapeutic effect of silver needle thermal conduction therapy on myofascial trigger point
Source: PLoS One. 2025 Sep 4;20(9):e0330592. doi: 10.1371/journal.pone.0330592 (PMC12410818; doi:10.1371/journal.pone.0330592)

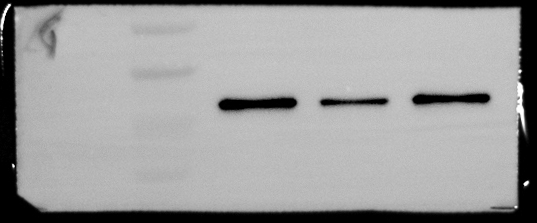

Supplement: S1 Raw images — (ZIP) [file pone.0330592.s001.zip › S1_raw_images/005-overlay[SIRT3].tif]

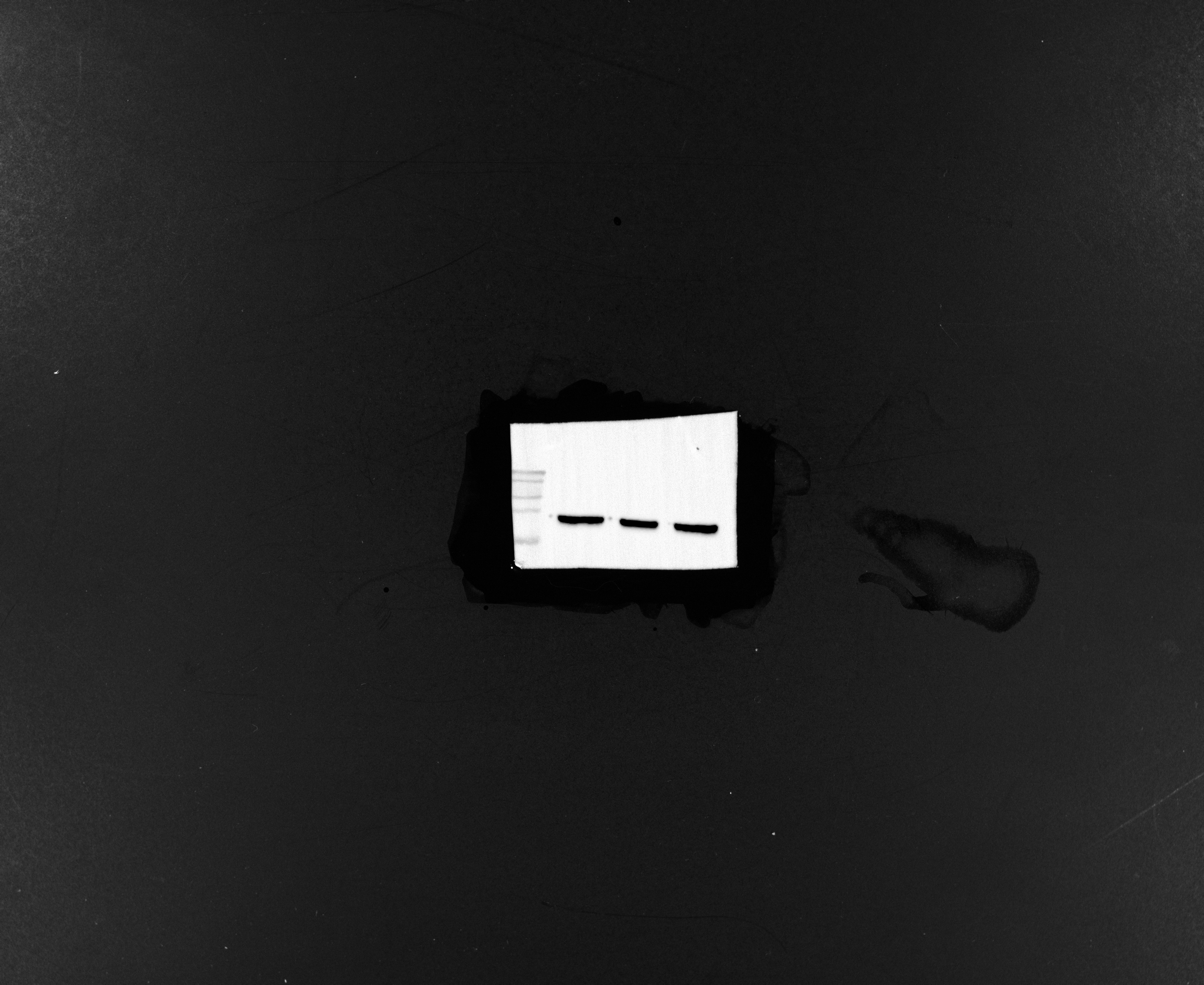

Supplement: S1 Raw images — (ZIP) [file pone.0330592.s001.zip › S1_raw_images/001-overlay[GAPDH].tif]

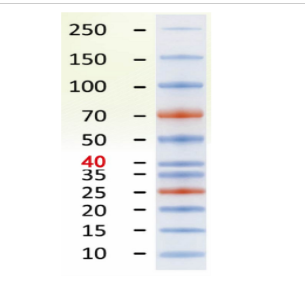

Supplement: S1 Raw images — (ZIP) [file pone.0330592.s001.zip › S1_raw_images/marker 4πÇü5.tiff]

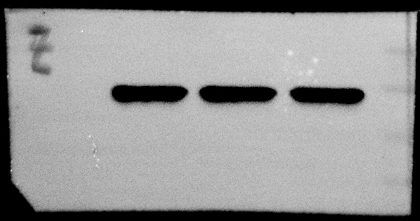

Supplement: S1 Raw images — (ZIP) [file pone.0330592.s001.zip › S1_raw_images/004-overlay[GAPDH].tif]

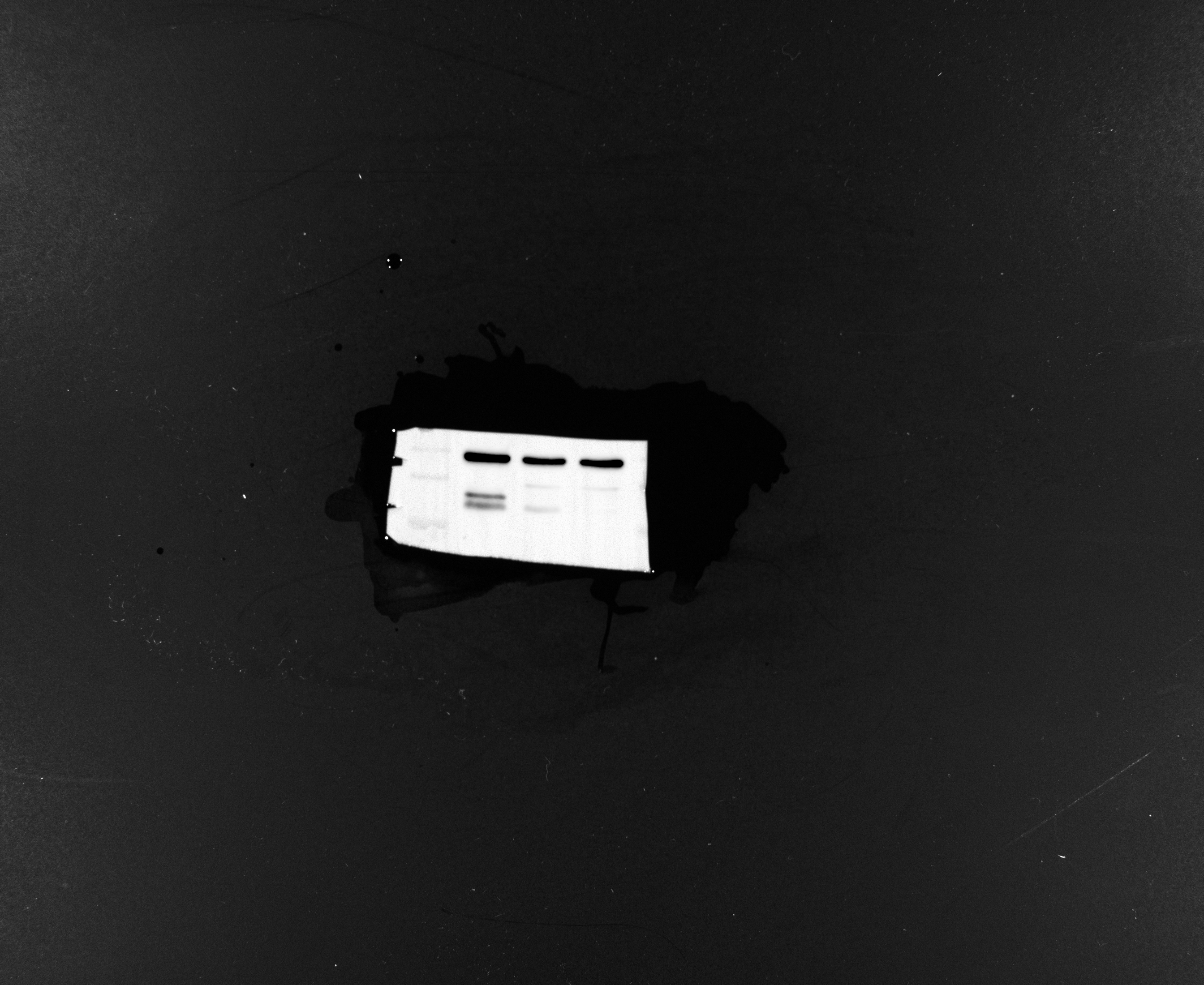

Supplement: S1 Raw images — (ZIP) [file pone.0330592.s001.zip › S1_raw_images/003-overlay[SIRT3].tif]

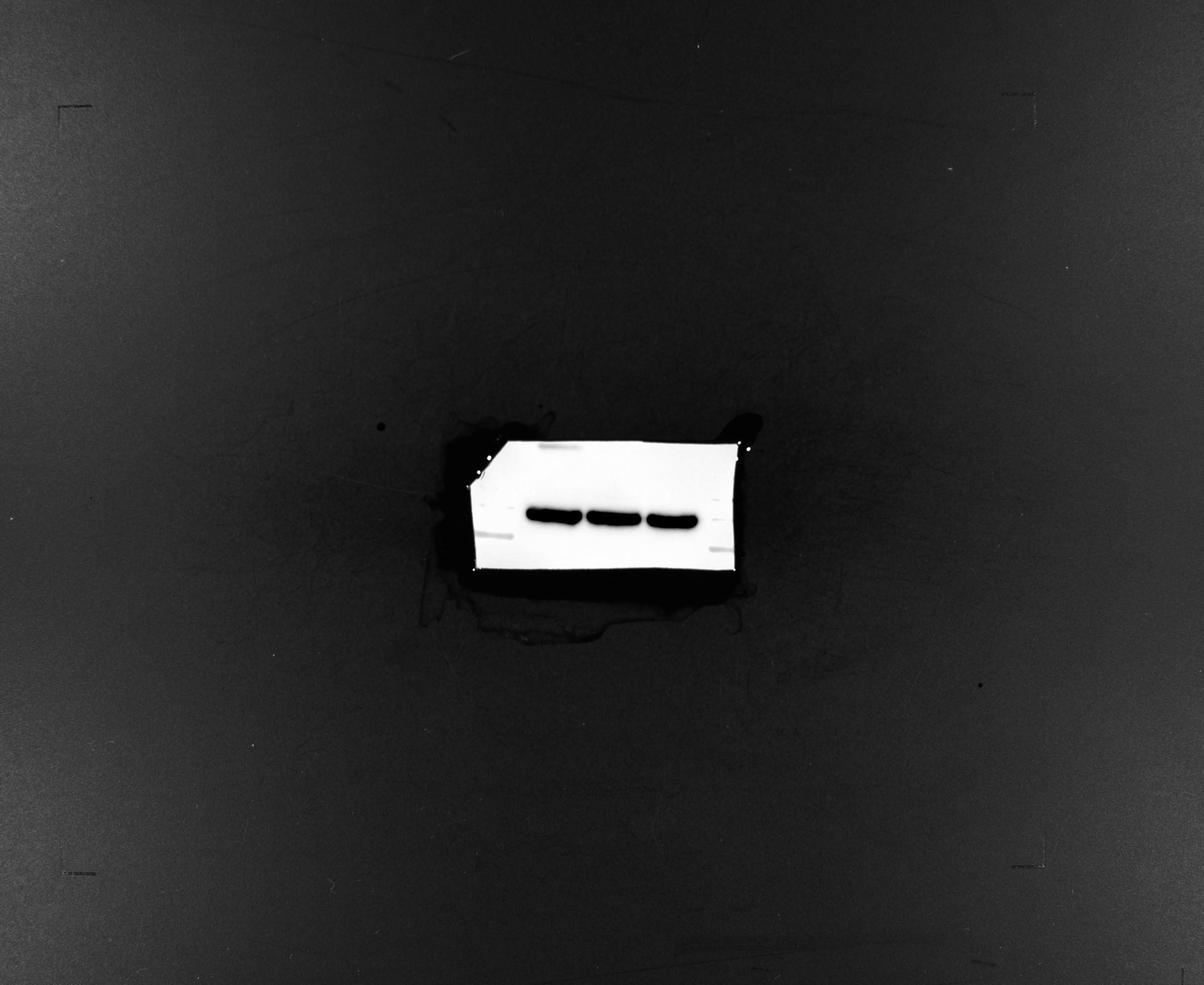

Supplement: S1 Raw images — (ZIP) [file pone.0330592.s001.zip › S1_raw_images/002-loverlay[GAPDH].tif]

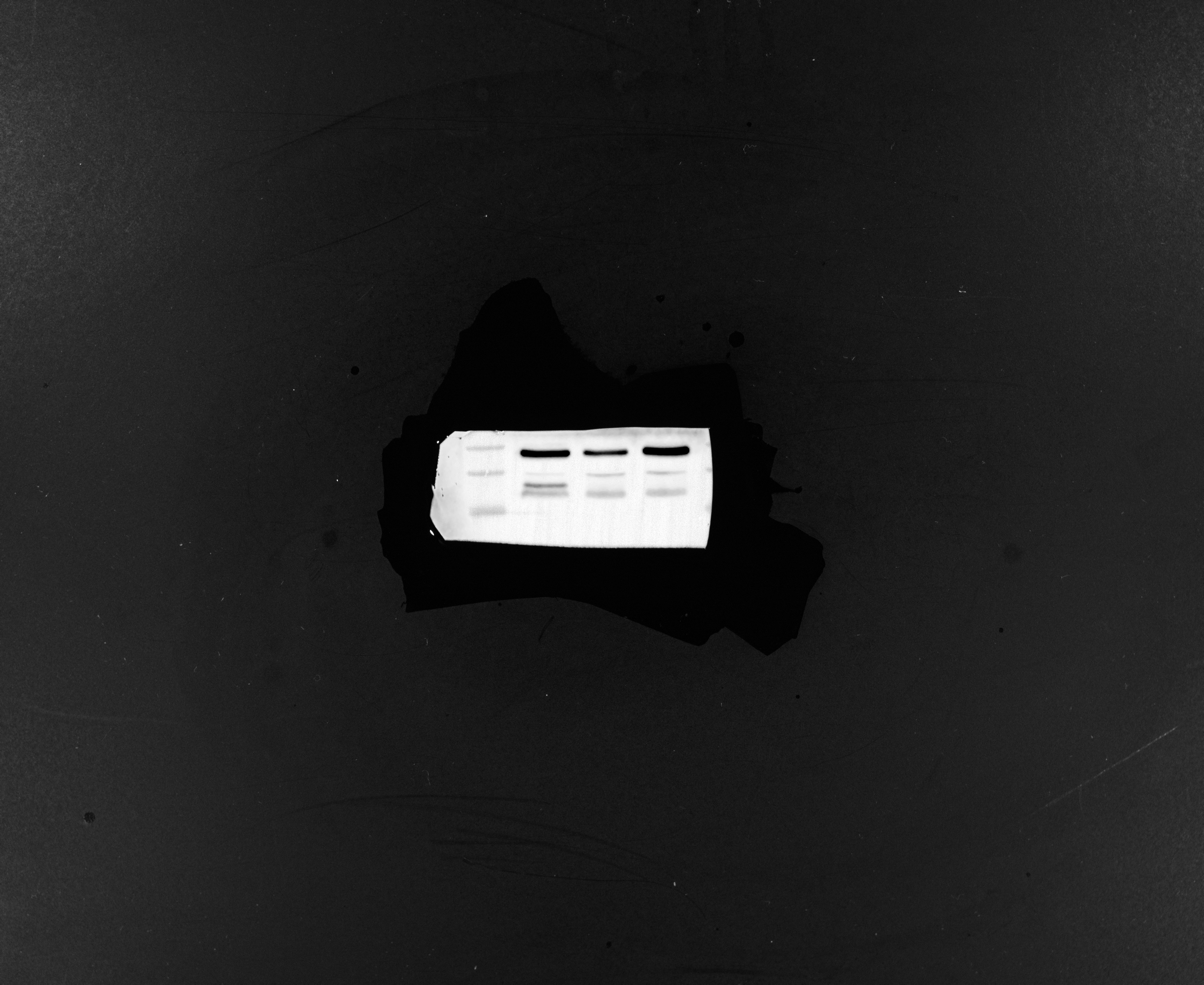

Supplement: S1 Raw images — (ZIP) [file pone.0330592.s001.zip › S1_raw_images/002-overlay[SIRT3].tif]

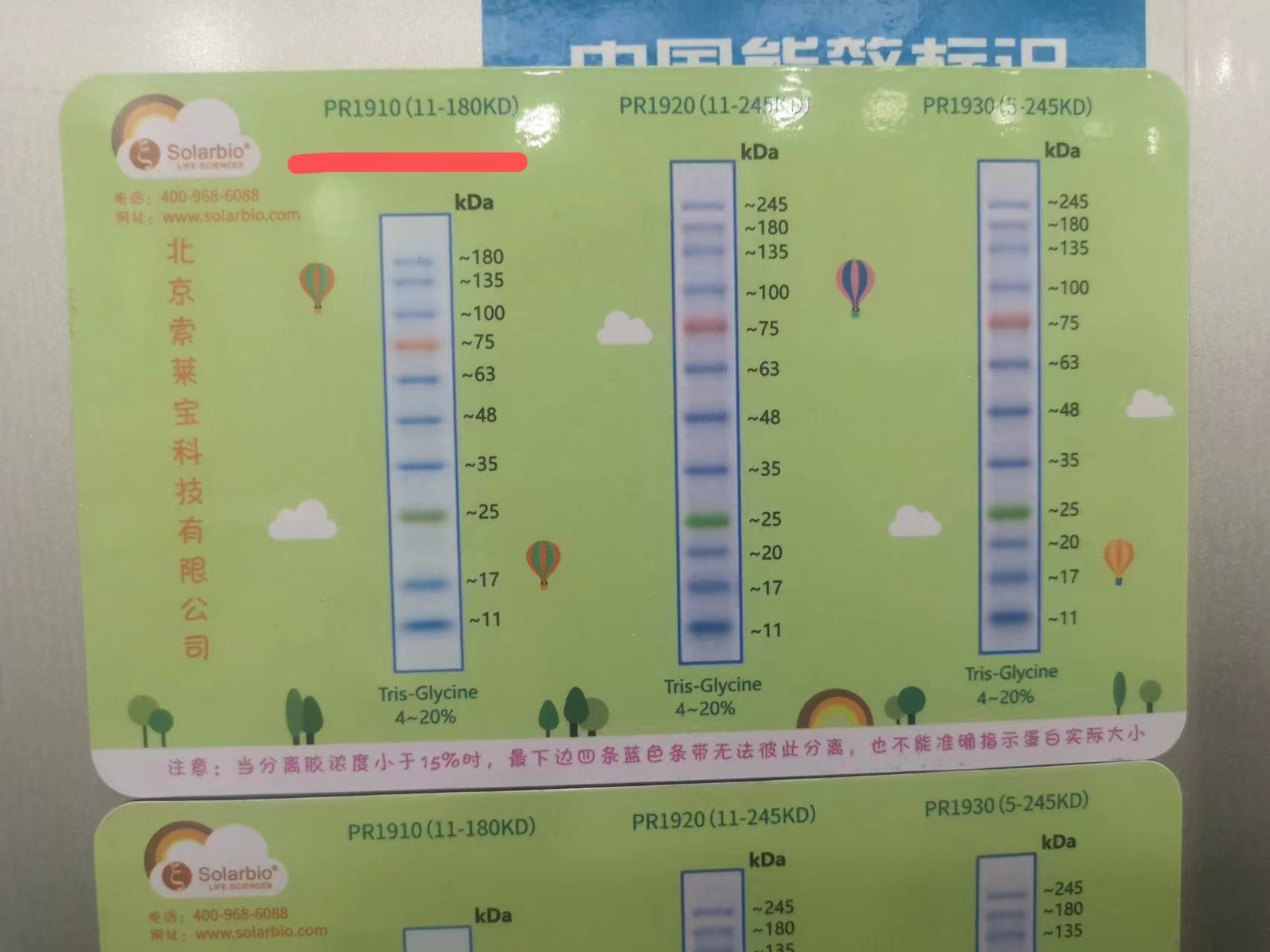

Supplement: S1 Raw images — (ZIP) [file pone.0330592.s001.zip › S1_raw_images/marker1πÇü2πÇü3 .jpg]

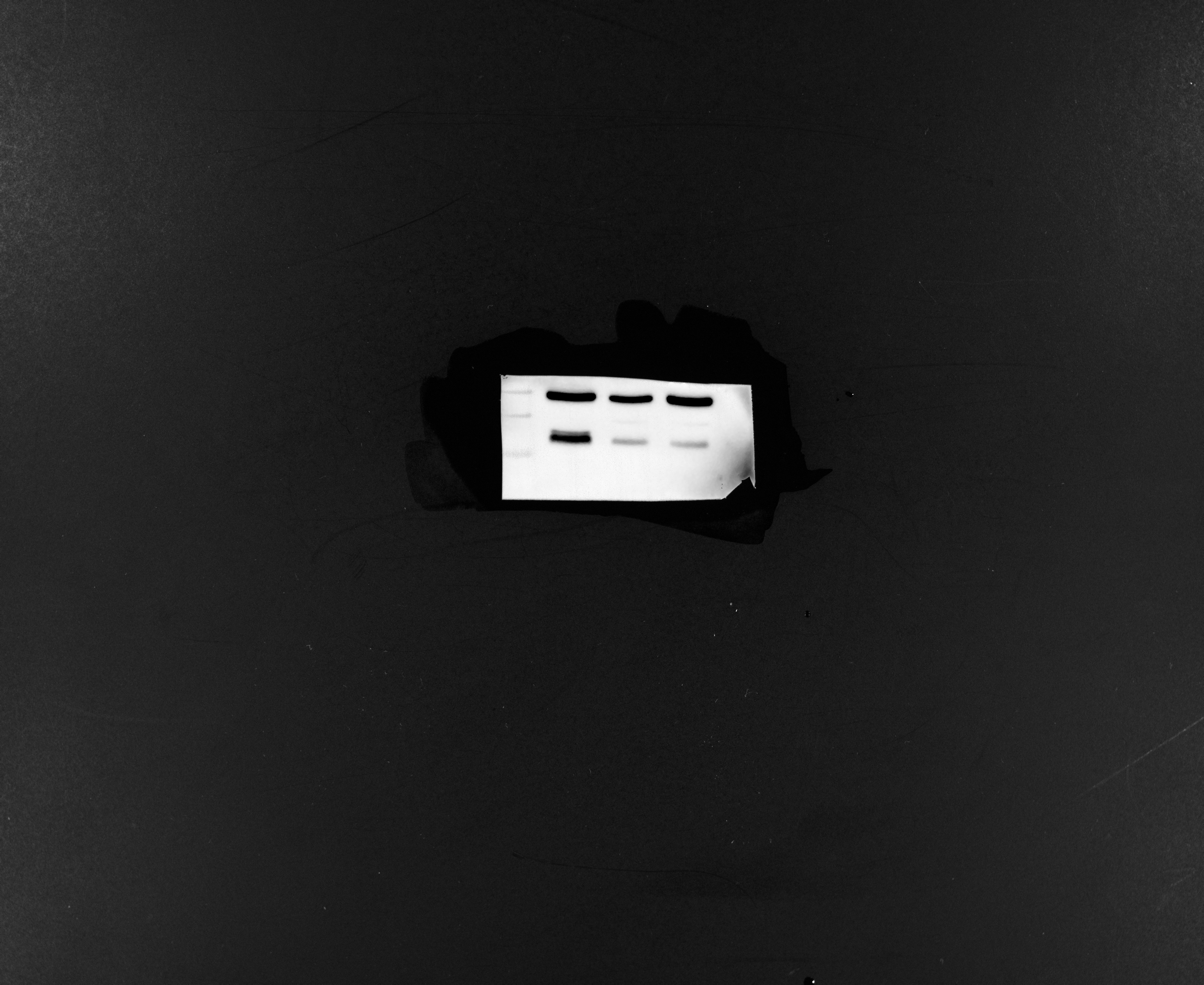

Supplement: S1 Raw images — (ZIP) [file pone.0330592.s001.zip › S1_raw_images/001-overlay[SIRT3].tif]

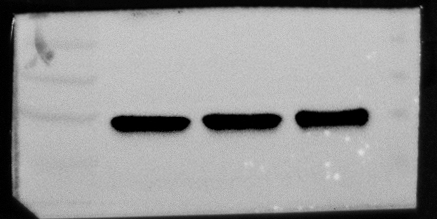

Supplement: S1 Raw images — (ZIP) [file pone.0330592.s001.zip › S1_raw_images/005-overlay[GAPDH].tif]

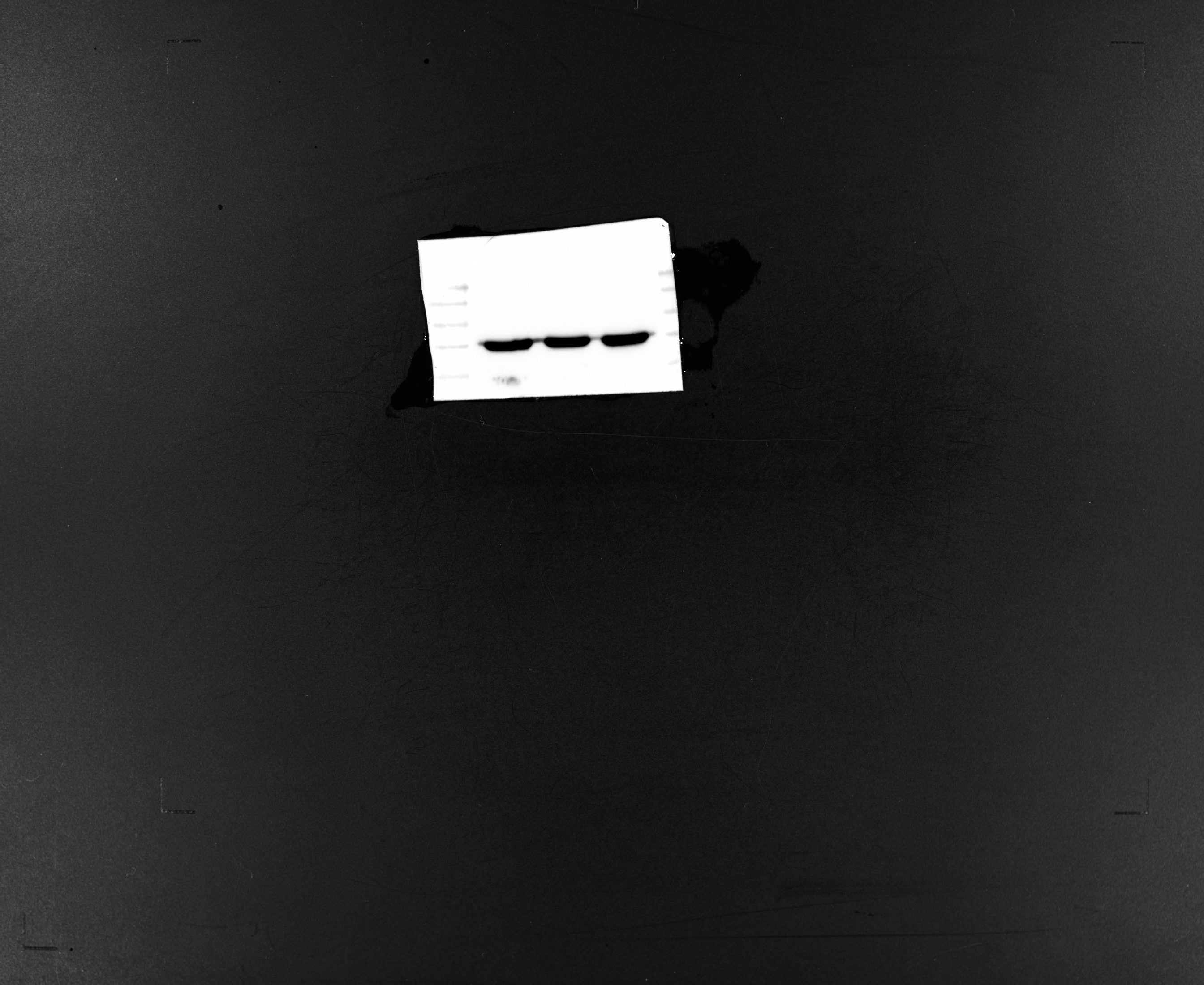

Supplement: S1 Raw images — (ZIP) [file pone.0330592.s001.zip › S1_raw_images/003-overlay[GAPDH].tif]

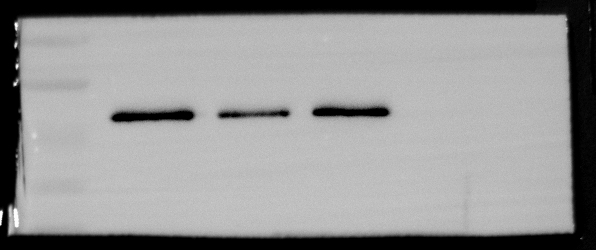

Supplement: S1 Raw images — (ZIP) [file pone.0330592.s001.zip › S1_raw_images/004-overlay[SIRT3].tif]
