# Supplementary material for: Quantitative evaluation of therapeutic effect of silver needle thermal conduction therapy on myofascial trigger point
Source: PLoS One. 2025 Sep 4;20(9):e0330592. doi: 10.1371/journal.pone.0330592 (PMC12410818; doi:10.1371/journal.pone.0330592)
Supplement: Attachment — Submitted filename: Response to Reviewers.docx [file pone.0330592.s003.docx]

We feel great thanks for your professional review work on our article. As you are concerned, there are several problems that need to be addressed. According to your nice suggestions, we have made extensive corrections to our previous draft, the detailed corrections are listed below.

To Reviewer #1: 
Points for Clarification:

Inconsistent Rat Numbers: You mention using 24 rats, divided into three groups of 5. However, the HE staining and TEM analyses mention 6 rats per group. Please standardize the number of rats per group for all analyses.
Reply: We were really sorry for our careless mistakes. We have made corrections as requested,as seen in page 5, line 93.

MPS Model: The description of the MPS induction method should be more detailed. Please specify the frequency and duration of the impacts, as well as the type and duration of the physical training imposed on the rats.
Reply: Thank you very much for your reminding. We have made corrections as requested,as seen in page 6, line 121 and 125.

Temperature Justification: You indicate that the needle temperature is 110°C, but that the skin surface temperature is 42°C. Explain the choice of these temperatures and how the skin temperature was controlled and maintained constant.

Reply: I apologize for causing confusion to you. Previous studies believe that the temperature of the silver needle body at the skin injection point should not exceed 42 ° C from the perspective of safety and effectiveness. Silver needle heating instrument conducts heat to the needle body by connecting with the tail end of the needle body. The temperature of the silver needle heating instrument is set to 110° C. After heat dissipation through the exposed needle body, silver needle body at the skin injection point does not exceed 42°C. It has been revised in manuscript, as seen in page 7,line 131.

Suggested Improvements:

SWE Analysis: Please specify the size and shape of the ROIs used for the SWE analysis, as well as the method of selecting these ROIs.

Reply: In the revised manuscript, the size and shape of the ROIs has been organized. It can be located on page 9，line 181. And we have described in more detail the methods for selecting these ROIs. The select method and the parameters of ROI are based on previous references，Lv H, Li Z, Hu T, Wang Y, Wu J, Li Y. The shear wave elastic modulus and the increased nuclear factor kappa B (NF-kB/p65) and cyclooxygenase-2 (COX-2) expression in the area of myofascial trigger points activated in a rat model by blunt trauma to the vastus medialis. J Biomech. 2018 Jan 3;66:44-50. The method of selecting these ROIs.

SIRT3 Interpretation: The increase in SIRT3 expression alone is not enough to conclude mitochondrial repair. Additional analyses of mitochondrial function would be necessary to support this conclusion.

Reply：We concur with you that more mitochondrial function results would be benefical.In this study, mitochondrial microstructure was used to evaluate mitochondrial function. We have re-written this this part. In the revised manuscript, we combined SIRT3 with mitochondrial microstructure to evaluate mitochondrial repair，as seen in page 19, line 310-324. Meanwhile, we have revised the manuscript as your recommendation.We have added a section in the discussion explaining the limitations of this paper，including the small sample size and the lack of confirmation of mitochondrial repair.Looking ahead, we will pay more attention to enriching experimental methods, such as increasing the detection of ROS, ATP and other indicators.

Limitations: It would be wise to include a section in the discussion to address the limitations of the study, including the small sample size and the lack of confirmation of mitochondrial repair.

Reply：We think this is an excellent suggestion. We have added this section to the last paragraph of the discussion，as seen in page 20 ,line 327-329.

To Reviewer #2:
Major Concerns

Targeting Accuracy of TrPs:
The study does not clearly address whether TrPs were precisely targeted. Imaging-based guidance (e.g., ultrasound) should be detailed to verify accurate targeting before and after the therapy.
Referencing established protocols for TrP detection using ultrasound, such as those by Bubnov et al., would improve the methodological section [Bubnov R.V. Evidence-based pain management: is the concept of integrative medicine applicable?. EPMA Journal 3, 13 (2012). https://doi.org/10.1186/1878-5085-3-13
Bubnov RV: The use of trigger point dry needling under ultrasound guidance for the treatment of myofascial pain (technological innovation and literature review). Lik Sprava. 2010, 5–6: 56-64.]

Reply: We sincerely appreciate your valuable comments. Silver needle heat conduction therapy has been used in clinical practice for decades, and it has good long-term curative effect. However, the evaluation criteria are subjective, mainly through VAS score. More objective evidence is lacking. Therefore, the purpose of this study was to quantitatively evaluate the efficacy of silver needle thermal conductivity therapy by T2mapping and STE, and to provide stronger evidence support for the treatment of MPS by this technology. In clinical practice, we determine the acupuncture site on the basis of anatomy by palpation of tension band and muscle twitching caused by acupuncture. Therefore, in order to avoid the influence of different manipulation methods on the results, we locate TrP by palpation combined with electromyography induced muscle twitching in animal experiments, without the use of image guidance for localization. We have refined and modified this content,which can be seen in page 8, line 174. Imaging is only used to assess the effectiveness of treatment. Your suggestions have provided us with a more optimized treatment plan, and we will carry out more accurate treatment according to your suggestions in the later stage, which may achieve more therapeutic effects and less pain.
US Imaging and Needling:
Ultrasound images should be included to showcase how TrPs were detected and evaluated pre- and post-treatment.
Dry needling, widely recognized as effective for TrPs, should be compared to silver needle thermal conduction therapy. This will establish the added value of the thermal conduction method.

Reply:We sincerely thanks for your careful reading and suggestion.We concur with you that more dry needling comparison would be benefical. However, due to the long modeling time (3 months) and the need to synchronize the existing three groups of animals which lead to animals waste, it is impractical to conduct additional experiments at present. On the other hand, the main purpose of this experiment is to conduct an quantitative evaluation of the curative effect of silver needle thermal conduction therapy, in order to provide more objective evidence for the silver needle thermal conduction treatment of MPS and a new treatment for MPS. Therefore, comparative studies with other treatments were not planned at the time of design. Looking ahead, we will put more emphasis on the efficacy of different treatments.
Mechanism of Action:

The manuscript does not adequately explain why silver needles were chosen. What unique mechanisms (chemical, mechanical, or thermal) contribute to their effects?
The absence of discussion regarding local twitch responses evoked during treatment is a missed opportunity to connect EMG findings with imaging data.
Reply:We think this is an excellent suggestion. In the revised manuscript, our explanation and discussion have been added,which can be found in page 3,line 45-49 and page19, line 302-309.

Title and Focus:
The title should explicitly reflect needling for TrPs instead of the generalized term "Myofascial Pain Syndrome."
Consider shortening the title to improve clarity and focus.

Reply: We feel great thanks for your professional review work on our article. We have revised our tittle.

Visualization:

The images do not sufficiently reflect TrPs as a target. Highlighting the precise region of TrPs in T2 mapping or STE images is essential.
Clear visualization would strengthen the study's impact and reproducibility.
Reply: We first palpate the muscle tension band and then identify the precise location of spontaneous electrical activity detected by EMG as TrP. Mark the location before performing a follow-up check.We revised it on the 174 line of page 8.

Mitochondrial Analysis:
While the mitochondrial findings are compelling, their relevance to the clinical outcomes should be elaborated. Does repairing mitochondrial damage directly correlate with pain relief or muscle function restoration?
Reply: According to your suggestion, we explained the relationship between Trp production and mitochondrial dysfunction in more detail, so as to conclude that silver needle heat conduction therapy may play a role in relieving pain by repairing mitochondria( page 19,line 310-324). However, it has not been proven that the functional repair of mitochondria can directly improve MPS pain. This is also the limitation of this experiment. Therefore, we have added this section to the last paragraph of the discussion,page 20, line 327-329.

Recommendations
Methods: Include a detailed description of ultrasound imaging protocols for TrP detection with references, and explain pre- and post-evaluation methods.

Reply: In the revised manuscript, the size and shape of the ROIs has been organized. And we have described in more detail the methods for selecting these ROIs, which can be seen in page 8, line 174 and page 9, line 181-184.

Comparative Analysis: Provide a robust comparison of silver needle therapy with dry needling or other mechanical stimulation methods.

Reply: Thank you for your suggestion. Your suggestion can more clearly explain the thermal effect of silver needle conduction therapy. For reasons such as time and animal waste, it is difficult for us to add this part in this article. In future studies, we will pay more attention to the comparison between different treatments.

Mechanisms: Discuss the rationale for silver needles and clarify the dominant mechanism (thermal vs. mechanical).

Reply:We have revised our manuscript, which can be seen in page 20, line 295-298,302-309.

Images: Add annotated imaging of TrPs for better visualization and reproducibility.

Reply: We have outlined the TrPs region with a dashed white line according to the comments.

Title Revision: Refine the title to emphasize needling for TrPs and its therapeutic implications

Reply: We have revised our title, which can be found in page 1, line 1.

While revising your submission, please upload your figure files to the Preflight Analysis and Conversion Engine (PACE) digital diagnostic tool, <https://pacev2.apexcovantage.com/>. PACE helps ensure that figures meet PLOS requirements. To use PACE, you must first register as a user. Registration is free. Then, login and navigate to the UPLOAD tab, where you will find detailed instructions on how to use the tool. If you encounter any issues or have any questions when using PACE, please email PLOS at <a href="mailto:figures@plos.org">figures@plos.org. Please note that Supporting Information files do not need this step.
