# Supplementary material for: Quantitative evaluation of therapeutic effect of silver needle thermal conduction therapy on myofascial trigger point
Source: PLoS One. 2025 Sep 4;20(9):e0330592. doi: 10.1371/journal.pone.0330592 (PMC12410818; doi:10.1371/journal.pone.0330592)
Supplement: Attachment — Submitted filename: Response to reviewers-2.docx [file pone.0330592.s005.docx]

Thank you very much for the valuable comments, which have been very helpful for our research and manuscript. We have responded and made modifications to your question, as follows.

Major Issues:

1. Justification for Silver Needle Thermal Conduction Therapy (SNTCT)

The manuscript should clearly define why the authors chose this method over conventional DN. The following aspects require detailed discussion:

Why introduce silver and heat to the needle when standard fine needles are already effective for trigger point inactivation?

What specific physiological or biomechanical advantages does silver provide in this context? If its antimicrobial properties or conductivity are relevant, these should be supported with evidence.

How does thermal conduction enhance the therapeutic effect beyond what is achieved with precise DN?

Is there prior research supporting the addition of these variables in needling therapy? If not, a stronger theoretical basis should be provided.

If silver is only used as a thermal conductor, why not use another material?

Reply：Thank you very much for your valuable comments. First of all, silver needles and dry needles are very different: 1, the treatment site is different: silver needles are based on the anatomical structure of the pain site, and the treatment is performed at the starting point, stopping point or shape of the muscle, while dry needles are treated according to the acupuncture point; 2. Different acupuncture depth: the depth of the silver needle reaches the periosteal, and the action site of the dry needle is relatively shallow; 3. Different thickness: the silver needle body is thicker, the diameter is about 1.1mm, the length is between 110-150mm, the dry needle diameter is about 0.3-0.5mm, the length is about 40-50mm ^[3, 4],^ the length and diameter of the silver needle is about 3 times that of the dry needle; 4. Different treatment times: silver needle thermal conduction therapy only requires a single treatment, while dry needle therapy usually requires regular multiple treatments, as seen in page 3, line 46.

Secondly, research indicates that dry acupuncture has poor long-term results ^[16, 17]^. For patients experiencing extreme pain, the impact is greatly diminished^[18]^. The clinical advantages of dry acupuncture in improving functional disorders and its follow-up effects remain unclear ^[19]^.When it comes to improving motor function and everyday life activities and reducing spasticity,warm acupuncture is better than electroacupuncture or acupuncture^[27]^. The clinical effect of fire acupuncture in treating spasticity after stroke is superior to that of traditional acupunctur^[28].^ The therapeutic effect of intensive moxibustion plus acupuncture is superior to that of simple acupuncture in improving symptoms of frozen shoulder in patients ^[29]^. It is suggested that thermal effect combined with mechanical effect is superior to mechanical effect alone. silver needle thermal conduction therapy has both thermal and mechanical effects, as seen in page 14, line 323 and page 14, line 341.

Silver has superior thermal conductivity compared to other metals, which is why silver needles are utilized^[30]^. However, because the application of silver needle thermal conduction therapy is equipped with heating equipment that has the function of temperature control, and we control the temperature of the skin injection point of the silver needle below 42℃, there is no need to worry about side effects like scalding caused by heating. The safety of this treatment has been validated by numerous Chinese literatures, as seen in page 14, line 346.

References:

[3]Ziaeifar M, Arab AM, Mosallanezhad Z, Nourbakhsh MR. Dry needling versus trigger point compression of the upper trapezius: a randomized clinical trial with two-week and three-month follow-up. J Man Manip Ther. 2019 Jul;27(3):152-161.

[4]Jo HR, Choi SK, Sung WS, Lee SD, Lee BW, Kim EJ. Thermal Properties of Warm- versus Heated-Needle Acupuncture. Evid Based Complement Alternat Med. 2022 Feb 28;2022:4159172.

[16]Rodríguez-Mansilla J, González-Sánchez B, De Toro García Á, Valera-Donoso E, Garrido-Ardila EM, Jiménez-Palomares M, González López-Arza MV. Effectiveness of dry needling on reducing pain intensity in patients with myofascial pain syndrome: a Meta-analysis. J Tradit Chin Med. 2016 Feb;36(1):1-13.

[17] Gattie E, Cleland JA, Snodgrass S. The Effectiveness of Trigger Point Dry Needling for Musculoskeletal Conditions by Physical Therapists: A Systematic Review and Meta-analysis. J Orthop Sports Phys Ther. 2017 Mar;47(3):133-149. [18]Gerber LH, Sikdar S, Aredo JV, Armstrong K, Rosenberger WF, Shao H, Shah JP. Beneficial Effects of Dry Needling for Treatment of Chronic Myofascial Pain Persist for 6 Weeks After Treatment Completion. PM R. 2017 Feb;9(2):105-112.

[19]Liu L, Huang QM, Liu QG, Thitham N, Li LH, Ma YT, Zhao JM. Evidence for Dry Needling in the Management of Myofascial Trigger Points Associated With Low Back Pain: A Systematic Review and Meta-Analysis. Arch Phys Med Rehabil. 2018 Jan;99(1):144-152.e2.

[27]Yang L, Tan JY, Ma H, Zhao H, Lai J, Chen JX, Suen LKP. Warm-needle moxibustion for spasticity after stroke: A systematic review of randomized controlled trials. Int J Nurs Stud. 2018 Jun;82:129-138.

[28]Qiu X, Gao Y, Zhang Z, Cheng S, Zhang S. Fire Acupuncture versus conventional acupuncture to treat spasticity after stroke: A systematic review and meta-analysis. PLoS One. 2021 Apr 9;16(4):e0249313.

[29]Gao L, Li X, Wang DB, Du ML, Xie J, Gao XY. [Clinical trial of treatment of frozen shoulder by intensive moxibustion plus acupuncture]. Zhen Ci Yan Jiu. 2019 Apr 25;44(4):297-301. Chinese.

[30]Lin L, Cheng K, Shen XY. [Analysis on the temperature-time curve in warm needling manipulation with acupuncture needles of different materials]. Zhongguo Zhen Jiu. 2019 Dec 12;39(12):1301-7. Chinese.

2. Mechanism of Action & Physiological Effects
   The study should clarify the hypothesized mechanisms through which silver and heat alter the effects of DN:
   The standard effectiveness of DN is largely attributed to precise intervention and eliciting localized twitch responses (LTRs) in trigger points. How do heat and silver modify this response?
   Could thermal conduction alter needle sensitivity, thereby influencing tactile feedback and the ability to localize trigger points?
   If heat increases circulation or pain modulation, was this measured or supported by prior research?
   Were adverse effects (e.g., risk of burns, altered tissue response) considered?

Reply:Thank you very much for your suggestion and we add further clarification to this section.Silver needles may exert therapeutic effects through thermal and mechanical effects. It has been reported in the literature that the needling action mechanically provides a localized stretch to shortened muscle segments and contracted cytoskeletal structures. This would restore the muscle segment to its resting length by reducing the degree of overlap between actin and myosin filaments^[20-22]^. Silver needle conductive therapy has a needling effect, which may exert its therapeutic effect by relaxing the muscles. Also, deep needling with dry needling has been shown to be more effective than shallow needling in treating pain associated with myofascial trigger points ^[23]^. The depth of action of silver needle thermal conduction therapy reaches the periosteum, which may be one of the mechanisms by which it exerts good therapeutic effects. At the same time, silver needle heat-conducting therapy may exert a thermal effect by transporting temperature to the body. The literature suggests that warm acupuncture is superior to electroacupuncture or moxibustion in reducing spasticity and promoting motor function and activities of daily living. When comparing warm acupuncture to electroacupuncture or moxibustion, the pooled results for spasticity effects and motor function were significant^[27]^. Compared to traditional acupuncture, fire acupuncture was more clinically beneficial in treating post-stroke spasticity^[28]^.Intensive moxibustion plus acupuncture was superior to simple acupuncture in improving patients' frozen shoulder symptoms ^[29]^. All of the above results suggest that the heat effect plays a role in improving spasticity.Combined with the result that silver needle thermal conduction therapy can improve muscle hardness, we speculate that silver needle thermal conduction therapy may have both mechanical and thermal effects. Its specific mechanism of action needs to be further explored，which can be seen in page14, line 330.

As the needling site, needle depth, and treatment modality (instrument heating, needle retention for 15-20 min, no repeated lifting and insertion, etc.) of silver needle are different from that of dry needle, there is no need to trigger a local twitch response and localize the trigger point during the implementation process. Trigger points are caused by localized muscle contracture, and silver needle thermal conduction therapy may improve muscle spasm through thermal effect, thus exerting analgesic effect instead of simply destroying trigger points directly, as seen in page 13, line 318.

Heat can increase circulation or pain modulation. Previous literature has reported that heat therapy promotes muscle relaxation, enhances blood circulation, and modulates injury receptors ^[24]^.An increased muscle temperature can initially cause substantial improvements in force production, faster rates of force generation, relaxation, shortening, and production of power output ^[25]^.Moxibustion heating dilates blood vessels, increases blood flow, and improves microcirculation ^[26]^，as seen in page 14, line 336.

The implementation of silver needle thermal therapy is equipped with a heating device that has the function of temperature regulation. We control the temperature of the skin entry point of the silver needle to below 42℃, which does not cause burns or necrosis of the muscle tissue.

Reference:

[20]Dommerholt J. Dry needling in orthopedic physical therapy practice. Orthop Phys Ther Pract. 2004;16 (3):15–20.

[21]Luke DR. Therapeutic needling in osteopathic practice: an evidence-informed perspective. Int J Osteopath Med. 2009;12(1):2–13.

[22]Dommerholt J, Del Moral OM, Gröbli C. Trigger point dry needling. In: Myofascial Trigger Points: pathophysiology and evidence-informed diagnosis and management. Jones & Bartlett Learning, 2009. p. 159–190.

[23]Kalichman L, Vulfsons S. Dry needling in the management of musculoskeletal pain. J Am Board Fam Med. 2010 Sep-Oct;23(5):640-6.

[24]Rossi R. Heat therapy for different knee diseases: expert opinion. Front Rehabil Sci. 2024 Jul 4;5:1390416. doi: 10.3389/fresc.2024.1390416. PMID: 39055174; PMCID: PMC11270809.

3. Study Design Considerations
   To isolate the true effect of silver and heat, the study should ideally include:
   A comparison between standard DN, silver-coated DN, and silver-thermal DN to assess individual contributions.
   A discussion on whether blinding was possible and how placebo effects were minimized.
   If no direct comparison was made, the authors should acknowledge this as a limitation and suggest future studies to address it.

Reply: Thank you very much for your valuable comments, which are very important for a deeper comprehension of the mechanisms of silver needle thermal therapy and may even reducing healthcare expenses. The study's shortcomings have been noted and incorporated in the discussion section (as seen in page15, line 365). In the subsequent experiments, we will add groups as per your suggestions to further clarify the thermal effect and needling effect of the silver needle.

4. Clinical Applicability & Cost-Benefit Considerations
If precise DN is already effective, does the added complexity and potential cost of SNTCT justify its use?
Are there specific clinical scenarios where silver thermal conduction needling would be superior to traditional DN?
Could this method introduce risks or complications that are not present with standard DN?

Reply: Long-term effect of dry needling is not good. In our clinical work we have found that silver needle thermal conduction therapy has definite and long term efficacy for MPS patients who are not well treated with dry needling. I myself am a patient of MPS in neck and shoulder, and the pain was relieved briefly after massage, oral medication and dry needling. By chance, I tried silver needle thermal conduction therapy, and after only one treatment, the pain was significantly relieved, and it has been nearly 10 years since then. As a result, I became interested in this folk therapy and conducted preliminary research. At the same time, silver needles can be sterilized and reused repeatedly, and although the input cost is high, it can be used for a long time.

Based on the good long-term efficacy of silver needles and the number of treatments in a single session, I believe that silver needle thermal conduction therapy is especially necessary.

The implementation of silver needle thermal conduction therapy is equipped with a heating device that has the function of temperature regulation, and we control the temperature at the point of entry into the skin of the silver needle to less than 42℃. The safety of this therapy is confirmed by a large number of literatures in our country.

5. Needling Precision & Local Twitch Responses (LTRs)
The therapeutic effectiveness of DN is highly dependent on precise needling technique and the elicitation of LTRs. The manuscript does not discuss whether silver-thermal needling affects this precision.
The authors should provide detailed descriptions of their needling protocol to ensure consistency with established DN methodologies, such as those described in [https://pmc.ncbi.nlm.nih.gov/articles/PMC3533862/].
If LTRs were monitored, this should be explicitly stated. If not, the authors should acknowledge that this is a limitation of the study.

Reply: In fact, the silver needle thermal conduction therapy does not locate based on trigger points, but rather arranges the needles according to the anatomy of the painful area and the tense zone, such as the starting point, ending point or course of the muscle in the painful area. A localized twitch response may be induced during needling. The needling and thermal effects may exert analgesic effect through other pathways than simply destroying the trigger point directly. The fact that it is not easy to precisely localize the trigger point, and it is difficult to implement even under ultrasound guidance, may be one of the reasons why the efficacy of dry needling is unstable and why ultrasound-guided dry needling has not been widely promoted. We describe this in more detail on page 14, line 326. Long-term efficacy monitoring was not performed, which is a shortcoming of this experiment and is described on page 15, line 367.

Minor issue:

Terminology consistency: Ensure that all references to the intervention (SNTCT, silver-thermal DN, etc.) are consistent throughout the manuscript.

Reply：We have harmonized this issue.

Literature review expansion: Include more references to existing studies comparing different needling techniques.

Reply：The differences between dry needle and silver needle have been explained in more detail in the manuscript，as seen in page13, line 318.

Clarify study limitations: If the study does not separate silver and heat effects, this should be acknowledged.

Reply：We recognize this limitation and account for it in the manuscript, as seen in page 15, line 367.

Conclusion:
The manuscript must provide a strong, evidence-based justification for incorporating silver and heat into DN. Additionally, the study design should either control for these variables separately or acknowledge this as a limitation. Addressing these concerns will significantly enhance the manuscript’s scientific and clinical relevance.

Reply：Your comments have been very helpful. In the manuscript we did not validate the thermal and pinprick effects of silver needle conductive therapy, and we have explained this limitation. And, we have added this part of the exploration in the new study and hope to report this issue more scientifically in future manuscripts. Thank you very much for your serious questions and scientific advice on the manuscript.

In addition, we have attached the following pictures, hoping to present the silver needle heat conduction therapy more intuitively. The following two pictures: The first one shows a patient with lumbar myofasciitis treated with silver needle heat conduction, and the second one is a comparison picture of silver needle and dry needle.
