# Supplementary material for: Quantitative evaluation of therapeutic effect of silver needle thermal conduction therapy on myofascial trigger point
Source: PLoS One. 2025 Sep 4;20(9):e0330592. doi: 10.1371/journal.pone.0330592 (PMC12410818; doi:10.1371/journal.pone.0330592)
Supplement: Attachment — Submitted filename: Response to reviewers-4.docx [file pone.0330592.s007.docx]

Thank you very much for your comments and suggestions on the manuscript, which have helped us present the content of the manuscript more precisely.While, we sincerely apologize for the inconvenience caused to you due to unclear identification of the modified content.We have responded and made modifications to your question, as follows.

Key Issues and Recommendations
1. Misrepresentation of Dry Needling
The authors' attempt to differentiate SNTCT from dry needling (DN) is not adequately supported and contains misleading claims. The response (Major Issue 1) suggests that DN is a superficial, acupuncture-point-based technique requiring repeated treatment sessions. This description does not reflect current clinical practice or research evidence.

Modern dry needling—particularly ultrasound-guided dry needling (US-DN)—enables precise targeting of myofascial trigger points (TrPs) under direct anatomical visualization. As shown in publications by Bubnov R. (Bubnov, 2013; https://www.liebertpub.com/doi/abs/10.1089/acu.2013.0973 https://linkinghub.elsevier.com/retrieve/pii/S0003496724543175, etc.), US-DN can be highly effective, reproducible, and minimally invasive, often requiring a single treatment without the need for thermal or material enhancements.

Additionally, the assertion that DN lacks long-term efficacy or acts solely through mechanical disruption without benefit is overly simplistic. DN protocols often elicit local twitch responses (LTRs), a known therapeutic mechanism in TrP inactivation. The role of SNTCT should be discussed as an alternative or adjunct, not a superior replacement, unless comparative data are provided.

Recommendation: The manuscript should be revised to accurately represent DN, particularly ultrasound-guided DN, and provide a balanced comparison. Please acknowledge that:

DN can be anatomically precise.

US guidance enhances safety and efficacy.

DN may not universally require repeated sessions.

Any claimed superiority of SNTCT should be tested in direct comparative studies.

Reply: We have made the necessary revisions to the above issues, as seen in line 340-345, page 14.

2. Study Design Limitations
   The lack of comparator groups (e.g., DN, silver-only, heat-only) prevents conclusions about which component(s) of SNTCT are responsible for observed effects. While acknowledged in the discussion, this limitation is fundamental and should be clearly emphasized in the conclusions.
   Reply：We have already emphasized this limitation as per your suggestion，as seen in page 15, line 364-367.

3. Clinical Translation
Given the strong animal data, the authors should discuss the translational roadmap for SNTCT, including safety, patient selection, cost implications, and potential for integration with ultrasound guidance.
Reply: Thank you very much for your valuable comments. Your suggestion is of great help to our subsequent research content. We may conduct further exploration in terms of economic costs and treatment guided by ultrasound. In fact, this technology has been applied in clinical practice for many years. Based on its definite long-term efficacy in clinical practice, our research group conducted some related studies on this therapy. In recent years, for the treatment of some specific areas such as the chest and back, ultrasound guidance has been adopted to enhance safety.

4. Terminology
Please ensure consistent use of terminology (e.g., "silver needle thermal conduction therapy" vs. "SNTCT" vs. "silver-thermal DN").
Reply: We once again checked the consistency of the "silver needle thermal conduction therapy" section in the manuscript.

5. Mitochondrial Mechanism
The link between mitochondrial repair and pain relief is plausible but remains speculative without direct measurement of functional mitochondrial output (e.g., ATP, ROS). This should be acknowledged more explicitly.
Reply: Thank you very much for your valuable comments. The limitation have been stated in the manuscript on page15, line 358-360.
